# A Systematic Review of the Role of Senescent Cells in Uterine Leiomyomas: Deciphering Molecular Pathways and Exploring Therapeutic Prospects

**DOI:** 10.1007/s43032-026-02075-x

**Published:** 2026-05-05

**Authors:** Shelby Howard, Akanksha Suresh, Morgan Bou Zerdan, Md Soriful Islam, Samya El Sayed, Rachel Michel, Mostafa Borahay, Sushma Nagaraj, Jennifer Elisseeff, Jude Phillips, Emily Joseph, Bhuchitra Singh, James H. Segars

**Affiliations:** 1 Department of Obstetrics and Gynecology, Louisiana State University Health Sciences Center, Baton Rouge, LA USA; 2 Johns Hopkins University School of Medicine, Baltimore, MD USA; 3 Faculty of Medicine, American University of Beirut, Beirut, Lebanon; 4 Division of Reproductive Sciences & Women’s Health Research, Department of Gynecology & Obstetrics, Johns Hopkins University School of Medicine, 720 Rutland Avenue Ross Research Building, Room 624, Baltimore, MD 21205 USA; 5 Department of Neurology, Brain Science Institute, Johns Hopkins University, Baltimore, MD 21231 USA; 6 Translational Tissue Engineering Center, Wilmer Eye Institute, Department of Biomedical Engineering, Johns Hopkins University, Baltimore, MD 21231 USA; 7 Department of Chemical and Biomolecular Engineering, Physical Sciences-Oncology Center, and Institute for NanoBioTechology, Johns Hopkins University, Baltimore, MD 21218 USA; 8 William H. Welch Medical Library, Johns Hopkins University, Baltimore, MD USA; 9 Nuffield Department of Women’s & Reproductive Health (NDWRH), Worcester College, University of Oxford, Oxford, UK

**Keywords:** Leiomyomas, Fibroid, Senescent cells, Senescence

## Abstract

Uterine leiomyomas (ULs) are prevalent benign tumors in women of reproductive age characterized by cellular senescence. Cellular senescence is a state of stable, irreversible cell cycle arrest characterized by discrete changes in cellular morphology and gene expression. This systematic review, following PRIMSA guidelines, evaluated the molecular pathways contributing to senescence in ULs and the use of novel therapeutic agents to target senescence. Two investigators independently screened and identified relevant articles written in English involving human subjects. Sixty-nine articles were identified; 11 studies met criteria. Multiple studies recognized a range of biomarkers of senescence in ULs including senescence associated beta galactosidase (SA-β-gal), senescent associated proteins (p16, p21, p14ARF), and telomere shortening. Key pathways such as AKT and p14ARF-TP53-p21, and genes such as *HMGA2* and *MED12* have been implicated in regulating the balance between tumor proliferation and growth arrest and senescence. However, the specific genetic and epigenetic mechanisms that induce and maintain senescence in ULs are not fully understood. There is growing interest in investigating whether senescent cells can be therapeutically targeted in ULs by senolytic agents that induce apoptosis, and senomorphic agents that modulate the senescence-associated secretory phenotype (SASP) to reduce its pro-tumorigenic effects. While limited, non-clinical data suggests this approach may be promising, further investigation is needed to establish their clinical efficacy in patients with ULs.

**Supplementary Information:**

The online version contains supplementary material available at 10.1007/s43032-026-02075-x.

## Introduction

 Existing evidence indicates that cellular senescence is a common feature in uterine leiomyomas (ULs), benign tumors that affect up to 80% of women [1–3]. Uterine leiomyoma significantly impact the quality of life for affected women [4] and contribute to an annual economic burden of over 42 billion USD in the US [5]. Cellular senescence is a state of stable, irreversible cell cycle arrest characterized by distinct morphological changes and alterations in gene expression. A key feature of cellular senescence is the senescence-associated secretory phenotype (SASP), that includes pro-inflammatory cytokines, growth factors, and matrix-remodeling enzymes [6, 7]. In normal physiologic states, cellular senescence plays an important role in suppressing tumorigenesis. Paradoxically, the senescent state has also been associated with promoting tumorigenesis through the downregulation of tumor suppression pathways and upregulation of pro-inflammatory SASP components that promote tumor growth, metastasis and resistance to therapy [8, 9]. In addition to ULs, cellular senescence is also commonly observed in benign tumors and premalignant lesions such as prostatic intraepithelial neoplasia, colon adenomas, and keloid lesions [10, 11].

Inducers of cellular senescence in ULs have been identified including the genes *WIPI1* and *SLITKR4* and the inactivation of the AKT pathway [12, 13]. Several biomarkers of senescence in ULs have also been recognized including senescence associated beta galactosidase (SA-β-gal), senescent associated proteins (p16, p21, p14ARF), and telomere shortening [14]. Despite these advances, several key questions remain. The exact molecular mechanisms that induce and maintain senescence in ULs, particularly the role of genetic and epigenetic factors, are not fully understood.

The clinical implications of targeting senescence in ULs for therapeutic purposes is an area that warrants further exploration. The current landscape of senescence therapies for cancer is characterized by a dual approach: inducing senescence to halt tumor growth and targeting senescent cells to prevent their pro-tumorigenic effects. Several conventional anticancer therapies such as chemotherapy and radiation exert their effects by inducing a stable cell cycle arrest in tumor cells that prevents further proliferation: a state of senescence [15, 16]. However, the senescence-associated secretory phenotype (SASP) poses a significant role in senescent tumor cells. The SASP secretome can be pro-inflammatory and thus promote tumor progression, metastasis, and therapy resistance [8].

This dual role of senescence necessitates additional therapeutic considerations to target senescent cells. Senolytics and senomorphics are two emerging classes of senescence-targeting therapies. While senolytics induce apoptosis in senescent cells, senomorphics target the SASP to mitigate its pro-tumorigenic effects without causing cell death [17]. Challenges in therapeutic targeting include the heterogeneity of senescent cells and the lack of universal biomarkers for their identification. This heterogeneity complicates the development of targeted therapies and the assessment of their efficacy [18, 19]. Additionally, the timing and combination of senescence-inducing and senescence-targeting therapies need optimization to maximize therapeutic benefits while minimizing adverse effects [15]. While senescence therapies are a promising area of research, several challenges remain in identifying and evaluating the therapies to improve clinical outcomes.

## Materials and Methods

### Information Sources

We searched publications in PubMed, Embase, Scopus and Web of Science from inception to November 2023. The review was conducted in accordance with Preferred Reporting Items for Systematic Reviews and Meta-Analysis (PRISMA) guidelines [20].

### Search Strategy

The data informationist (EJ) designed a list of terms related to uterine leiomyoma and senescence including “cellular senescence” OR “senescence” OR “senescent” OR “senescent cell*” OR “cell aging” OR “cellular aging” OR “cell ageing” OR “cellular ageing” OR “immunosenescence” OR “senesce” AND “Leiomyoma” OR “leiomyoma” OR “leiomyomas” OR “leiomyomata” OR “leiomyomatas” OR “fibromyoma” OR “fibromyomas” OR “myoma” OR “myomas” OR “myomatosis” OR “fibroid” OR “fibroids” AND “uterus” OR “uterine” OR “uteri” OR “myometrium” OR “Myometrium” OR “ULM”. A detailed list of the search strategy used is included in Appendix Item 1.

### Eligibility Criteria

We included English-language publications on senescent cells in relation to leiomyoma and myometrium. Studies were limited to human subject research, and study types included randomized control trials and observational studies. Review articles, animal subject studies, case reports, and non-English publications were excluded. A detailed eligibility criteria is included in Appendix item 2.

### Study Selection

After initial search strategies were executed, two independent reviewers (SH, MBZ, AS, SES) screened titles and abstracts using the eligibility criteria. Conflicts in the designation of studies inclusion were resolved by discussion, and any discrepancies were resolved by the senior author (BS or JHS). After completing the title and abstract screening, full-text screening was conducted similarly using the same inclusion and exclusion criteria. Observational studies and basic science research that demonstrated an association between senescence and leiomyoma, were included in the review.

### Assessment of Risk of Bias

Study quality was assessed by two reviewers independently, and any discrepancies were resolved by the senior author. Observational studies were determined to be good, fair, or poor quality based on the Newcastle-Ottawa Scale [21]. Quality was determined by the assessment of selection, comparability, and outcome categories.

### Data Extraction and Analysis

In this systematic review, the data extraction process involved reviewers (SH, MBZ, AS, SES) extracting relevant information such as study design, methods, results, demographics, experimental procedures, and the specific genes involved. The obtained results were then systematically arranged based on fibroid mutations, genetic pathways involved in senescence and possible treatment strategies. Extracted data was analyzed descriptively.

## Results

**Fig. 1:**
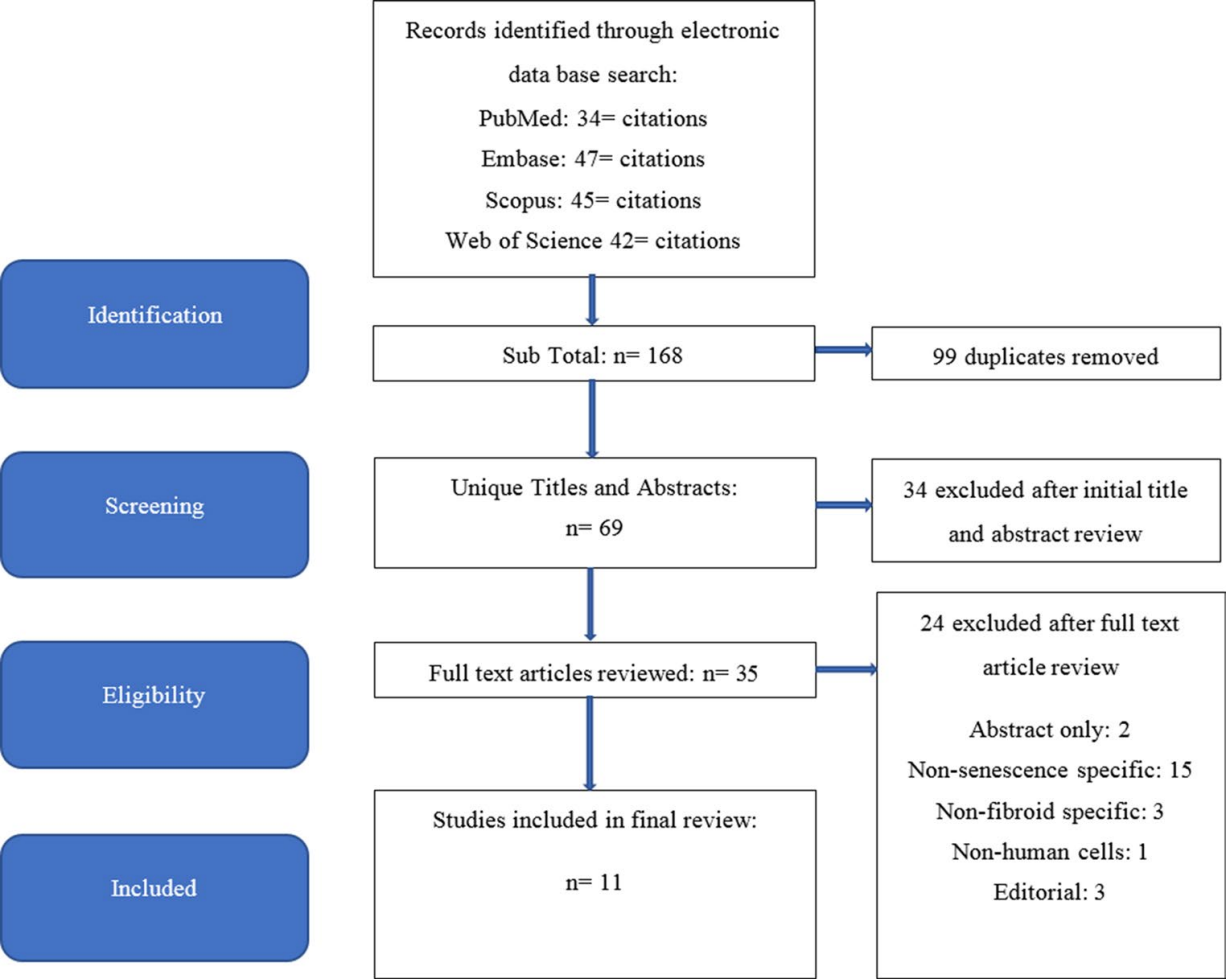
PRISMA flow chart of article identification, retrieval, review, and inclusion of studies

The initial search yielded 34 articles in PubMed, 47 in Embase, 45 in Scopus and 42 in Web of Science. After duplicates were removed, 69 unique articles underwent initial title and abstract review. Thirty-five articles were considered for full-text review (Fig. 1). Eleven studies met complete inclusion criteria and were included in this systematic review. All the studies included were observational. Nine of the studies were of good quality, one fair, and one poor when elevated using the Newcastle Ottawa Scale to assess risk of bias. However, all eleven studies were included due to limited availability of data on the topic. Extracted data were evaluated based on patient demographics, fibroid mutations, genetic pathways involved in senescence and possible treatment strategies as listed in Table 1.

**Table 1:** Summary of data on studies evaluating senescence in leiomyomas*

Author	Country	Sample Size	Association studied	Results	Race	Age (Mean)	Size ULM (cm)	Genetic data	Location of ULM	Route of Tissue collection	Senolytic or senomorphic evaluated	NOS
Xie (Oct 2018)	USA	178	gene mutation and ULM growth	ULM compositions Δ mutation. ER and PR expressed all ULM. HMGA2 ^ AKT signaling and larger tumors inhibiting HMGA2 increased p16 and p21.	—	MED12 46.1 ± 6.2 HMGA2 45.6 ± 5.7 FH 37.7 ± 1.7	MED12 6.7 ± 0.8 HMGA2 11.2 ± 2.4 FH 8.5 ± 0.8	₹	—	H	—	Good
Xie (Dec 2018)	USA	28	Pathways of inducing senescence	CS is induced both replicative and through stress. Genes identified in both replicative and stress WIPI1 and SLITKR4.	yes	45.1 ± 4.6	9.4 ± 4.8	₹	—	H	Yes ∞	Good
Xu 2014	USA	> 10 ULM	AKT inhibition	MK-2206 = ^ ROS, ^ microRNA, miR-182 and several senescence-associated genes	—	—	—	—	—	M or H	—	Good
Markowski et al. (2011)	Germany	28 ULM and MM	genes in CS on ULM growth and tx MDM2 inhibitor	HMGA2 inverse relation of p14Arf. nutlin-3 ^ BAX. p14Arf triggers CS and apoptosis	—	51.4 ± 6.8	3.3 ± 3.0	×	—	—	Yes ∞	Good
Xu 2018	USA	28	miRNAs in CS in 3D spheroid culture systems	*miR-29b*, *miR-181a*, *miR-182*, and *miR-200c* overexpression ^ CS. miR-181a and miR-182 = CS in ULM	yes	43.9 ± 4.4	9.4 ± 4.8	—	—	H	Yes ∞	Good
Laser et al. (2010)	USA	86 ULM 14 pts	CS in symptomatic fibroids.	58% SA-b-Gal + in > 10% of the tumor volume. ^ CS in small fibroids and older-aged women.	yes	48.6 ± 1.41	3.6 ± 2.2	—	Yes	M or H	—	Good
Markowski et al. (2012)	Germany	52 ULM + MM 36 pt	MDM2 inhibitors	p19Arf ULM > MM ULM are more sensitive to nutlin than MM	—	49.1 ± 8.3	3.8 ± 2.7	×	—	—	Yes ∞	Good
Markowski et al. (Aug 2010)	Germany	36 ULM 8 MM	HMGA2 p16(ink4a) and p19ARF	p19Arf ULM > MM HMGA2, p19Arf, and CDKN1A correlated with the size of the tumors	—	—	—	₹	—	—	—	Fair
Markowski et al. (Oct 2010)	Germany	16	HMGA2 expression in vivo vs. in vitro	HMGA2 inverse relation of p19Arf. Early CS in vitro cell cultures.	—	—	—	×	—	—	—	Good
Oh (2021)	Korea	18	Telomere length	Shorter telomeres ULM vs. MM.	—	45.7 ± 3	—	—	—	M or H	—	Good
Dickson et al. (2013)	Canada	*N* = 164 *n* = 10	SIRT1 in mesenchymal neoplasms	SIRT1 expression in 90% ULM.	—	—	—	—	—	—	Yes Δ	Poor

*CS* Cellular Senescence, *M* Myomectomy, *MM* Matched myometrium, *ULM* Uterine leiomyoma, *H* Hysterectomy, *NOS* Newcastle-Ottawa Scale

—: not reported

Δ: senomorphic: SIRT1

∞: senolytic: ABT263, Nultin-3

×: Karyotype listed

₹: Mutations listed

* all are observational studies

###  Evaluation of Senescence in Leiomyomas

The key biomarkers used to detect senescent cells in uterine leiomyomas (ULs) include SA-β-gal expression, expression of senescence-associated proteins p16, p21 and *HMGA2*, and telomere shortening, as summarized in Table 2.

**Table 2:** Results of tests for cellular senescence in leiomyomas

Author	Primary Evaluation of Senescence			Secondary Evaluation of Senescence	
	Evaluation of Senescence	Quantified	Timing of stain	Evaluation of Senescence	Quantified
Xie (Oct 2018)	SA-B-gal	numbers not given but reports intensity on graph	overnight	p16 and p21	Western blot
Xie (Dec 2018)	SA-B-gal	numbers not given but reports intensity on graph	overnight	p16 and p21	Western blot
Xu 2014	SA-B-gal	numbers not given but reports intensity on graph	16 h	p16, p21 and p53	RT PCR
Markowski et al. (2011)	SA-B-gal	count total number of cells	24 h	CDKN1A- p21	RT PCR
Xu 2018	SA-B-gal	1.5-fold increase in cellular senescence	16 h	—	—
Laser et al. (2010)	SA-B-gal	% of tumor cells that were positive	12–16 h	—	—
Markowski et al. (2012)	p21	Quantitative RT-PCR	—	—	—
Markowski et al. (Aug 2010)	p21	Quantitative RT-PCR	—	—	—
Markowski et al. (Oct 2010)	p19ARF	Quantitative RT-PCR	—	—	—
Oh (2021)	telomeres	—	—	—	—

— : not reported

Laser et al. [22] demonstrated that a significant proportion of ULs exhibit senescent changes: SA-β-gal expression was observed in greater than 10% of the tumor volume in 58% of the tumors studied. Additionally, the study found evidence of reduced proliferative activity via elevated levels of let-7 microRNAs (let-7c, let-7d, and let-7f-2) and a low Ki-67 index in senescent ULs. SA-β-gal was the most widely used marker of senescence, utilized in half of the articles surveyed in this review. However, an important limitation of SA-β-gal as a senescence marker is that it is not specific to senescent cells, but rather marks quiescent cells, and cells under stressors such as starvation and oxidative stress [23]. Thus, using SA-β-gal in conjunction with other markers such as Ki-67 and p16^INK4a may more accurately identify senescent cells [22].

Markowski et al. [24] evaluated the role of p14ARF in the growth of ULs. Their findings revealed that ULs express significantly higher levels of p14ARF mRNA compared to normal myometrium, with the greatest increase seen in ULs with 12q14-15 rearrangements, compared to those with other cytogenic changes. The expressions of p14ARF and p21 were also significantly correlated, suggesting that p14ARF triggers senescence rather than apoptosis in these tumors. Thus, the p14ARF-p53-p21 pathway plays a crucial role in the growth control of ULs and the expression of these genes may be useful biomarkers of senescence.

Furthermore, telomere shortening and the expression of telomere-related proteins such as TRF1 and TRF2 have been implicated in the senescence of ULs. Telomere shortening is closely linked to cellular senescence as it triggers cell cycle arrest when telomeres reach a critical length, activating pathways that lead to senescence or apoptosis [25]. Thus, telomere shortening serves as both a marker and mechanism of cellular senescence. Oh et al. [25] reported shorter telomeres in leiomyoma tissues compared to adjacent normal myometrium, suggesting active proliferation and subsequent senescence.

Overall, these biomarkers collectively provide insights into the cellular mechanisms underlying senescence in uterine leiomyomas and may serve as potential targets for therapeutic interventions aimed at modulating fibroid growth.

### Variability of Senescence Expression in Leiomyomas

#### Variability of Senescence Based on Age

One study suggests that uterine leiomyomas (ULs) in older women have more marked senescence profiles. Laser et al. [22] found that a higher expression of senescence-associated beta-galactosidase (SA-β-gal) was observed in smaller fibroids and in older-aged women. Thus, cellular aging processes in ULs may become more pronounced in the older age group.

#### Variability of Senescence Based on Genetic Mutation

Overexpression of *HMGA2* is associated with increased tumor growth and angiogenesis via the AKT signaling pathway in uterine leiomyomas [26]. The 12q14 ~ 15 chromosome translocation has been associated with overexpression of the *HGMA2* gene [27, 28], with one study noting greater than a hundredfold increase compared to normal myometrium. Silencing *HMGA2* in leiomyoma cells leads to downregulation of the AKT pathway and upregulation of p16 and p21, which in turn induces cellular senescence [26]. This suggests that *HMGA2* is a key player in the balance between promoting tumor proliferation and growth arrest and senescence.

p14ARF expression in ULs plays an important role in cell cycle regulation. The p14ARF protein, encoded by the *CDKN2A* locus, works by stabilizing p53 through *MDM2* inhibition. This subsequently leads to cell cycle arrest in G1 and G2/M phases and promotes apoptosis in response to oncogenic stress [29, 30].

The directionality of the relationship between *HMGA2* expression and p14ARF is likely complex and context dependent. One study by Markowski et al. [28] noted that *HMGA2* overexpression was correlated with decreased p14ARF expression. Another study by the same group [30] found the opposite: *HMGA2* overexpressing 12q14 ~ 15 ULs had 16.6-fold increased expression of p19ARF, the murine analog of p14ARF [31]. Significant correlations between p19ARF and p21 levels were additionally observed [30]. In another study, the use of an *MDM2* inhibitor, a p14ARF mimetic, induced cellular senescence and increased the expression of both p21 and the apoptosis regulator protein BAX [28]. Overall, these studies support the role of p14ARF in promoting cellular senescence and apoptosis in response to oncogenic stress via the P14ARF-p53-p21 pathway.

*MED12* mutations are prevalent in ULs and are associated with altered cellular pathways that influence tumor growth and senescence. Leiomyomas with *MED12* mutations often exhibit distinct molecular characteristics compared to those with *HMGA2* overexpression. ULs with *MED12* mutations had varied cellularity with prominent extracellular matrix and limited vasculature whereas ULs with *HMGA2* mutations had increased cellularity and vasculature, and an overall larger tumor size [26]. However, the co-occurrence of *MED12* mutations and *HMGA2* overexpression has been observed, suggesting intricate interactions between these genetic alterations [32].

### Role of the AKT Pathway in Leiomyoma Senescent Cells

The AKT pathway plays a crucial role in the regulation of cellular senescence in uterine leiomyomas (ULs). Inactivation of AKT induces cellular senescence in ULs, as demonstrated by several studies.

Xu et al. [12] showed that inhibition of AKT using the allosteric inhibitor MK-2206 led to increased levels of reactive oxygen species (ROS), upregulation of microRNA miR-182, and activation of several senescence-associated genes such as *CDKN2A*,* TP53*,* CDKN1A*, and *GLB1* (which makes the protein SA-β-gal). Thus, AKT inhibition led to a state of stress-induced premature senescence [12]. Further supporting this finding, Xie et al. [13] utilized an ex vivo spheroid model to show that AKT inhibition by MK-2206 was followed by cells undergoing stress-induced senescence, characterized by upregulation of ROS and hypoxia-related genes [13]. The study also identified genes such as *WIPI1* and *SLITKR4* that were associated with senescence in ULs.

The expression of the AKT pathway is intricately tied to that of *HMGA2*. *HMGA2* overexpression in ULs activated AKT pathway, and conversely, inhibition of *HMGA2* led to the downregulation of the AKT pathway and upregulation of senescence markers p16 and p21, resulting in cellular senescence [26]. Another study highlighted the role of *HMGA2* in mediating senescence, through demonstrating its co-localized with senescence-associated heterochromatin foci [12].

**Fig. 2:**
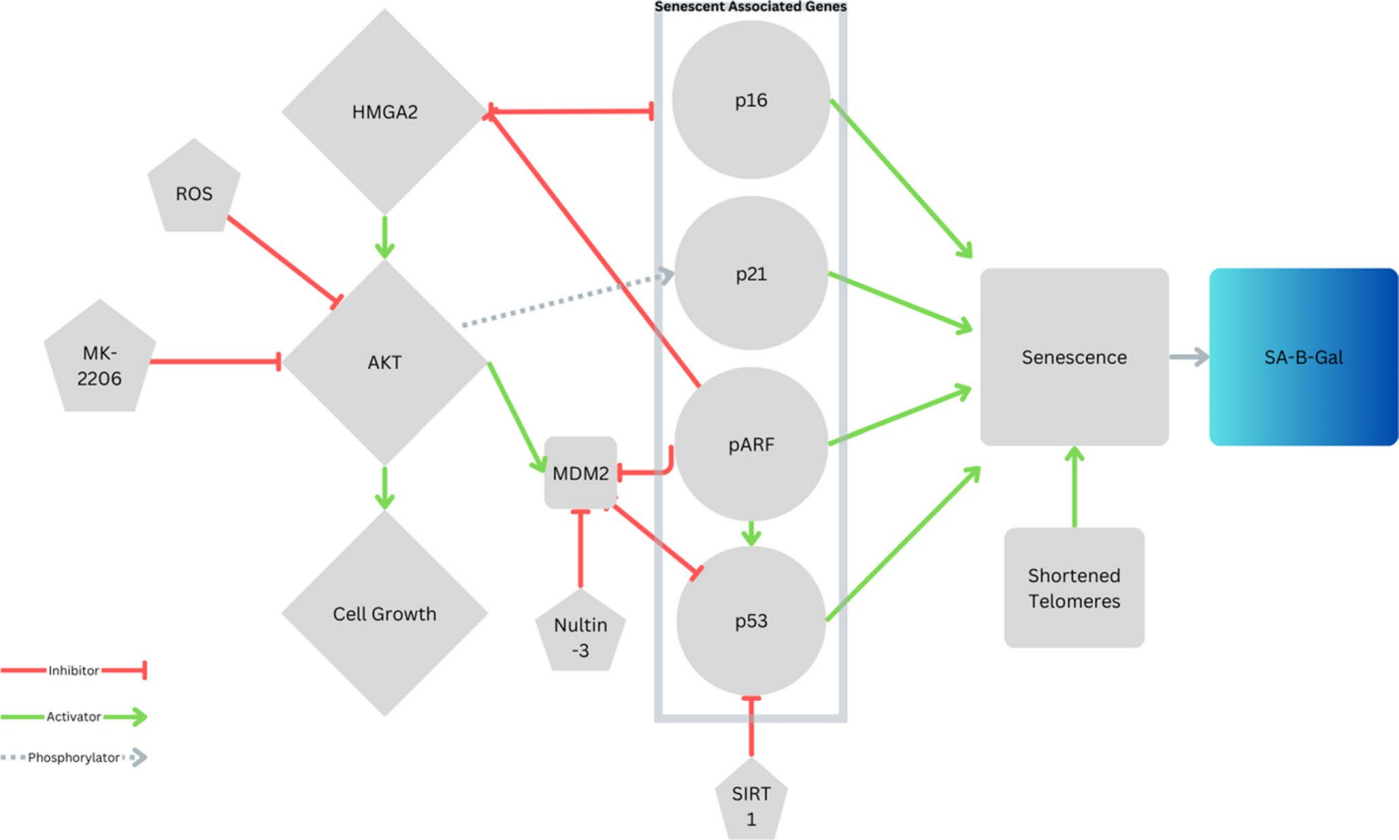
Pathways associated with senescence evaluated through this systematic review. AKT and HMGA2 are the main drivers of leiomyoma growth. When inhibited increase senescence associated genes, increased expression of p16, p21, pARF or p53. Can AKT pathway can be inhibited through MK-2206 and ROS. Nultin-3, a senolytic, works by inhibiting MDM2 allowing the cell to activate p53. SIRT-1 a senomorphic, inhibits p53 avoiding cellular senescence

In summary, the AKT pathway is crucial for the proliferation and survival of ULs, and its inhibition can induce cellular senescence through mechanisms involving ROS, microRNAs, and specific senescence-associated genes, as summarized in Fig. 2.

### Senescent Cells as a Potential Therapeutic Target in Leiomyomas

Senescent cells in ULs (ULs) exhibit a distinct signature characterized by the upregulation of reactive oxygen species (ROS), hypoxia-related genes, and SA-β-gal, as well as the senescence-associated secretory phenotype (SASP). This signature has the potential to direct therapies to manage ULs.

SA-β-gal expression serves as a biomarker for identifying senescent cells and evaluating therapeutic efficacy. Laser et al. [22] demonstrated that nearly 60% of leiomyomas exhibit pronounced SA-β-gal expression, which may correlate with the variation in tumor growth rates and help direct targets to prevent fibroid growth.

The upregulation of ROS and hypoxia-related genes in senescent ULM cells suggests that cellular senescence is induced and maintained by oxidative and hypoxic stress. Targeting these pathways can potentially disrupt the survival of senescent cells. Senolytics are therapeutic agents that induce cell death in senescent cells. The use of senolytic agents like ABT263 (a Bcl-2 homology domain 3 mimetic that inhibits the Bcl-2 pathway) has been shown to significantly reduce the number of senescent cells in UL spheroids by inducing apoptosis in these cells [13, 33]. This agent, ABT263, is currently in Phase II clinical trials for relapsed small cell lung cancer [34]. These studies also noted an overall increase in apoptosis in leiomyoma cells beyond just the senescent subtype [13, 33], suggesting a broader role for senolytic agents such as ABT263 in UL tumor management.

Another emerging senolytic is nutlin-3, a mouse double minute 2 homolog (MDM2) protein inhibitor that upregulates the p53 tumor suppressor pathway. Nutlin-3 has been shown to induce both apoptosis and senescence in a dose-dependent manner through significantly upregulating Bcl2-associated X protein (BAX) and p21, critical markers of the intrinsic apoptosis pathway and downregulating proliferation as operationalized by decreased Ki67 expression [24]. Interestingly, leiomyoma tissue was found to be more sensitive to the apoptotic effects of nutlin-3 compared to surrounding myometrial tissue [24], as seen by increased expression of the apoptotic marker p53 in treated leiomyoma vs. treated myometrium. Thus, the senolytic agent nutlin-3 may selectively target ULs with relative preservation of normal myometrial tissue.

The senescence-associated secretory phenotype (SASP) in ULs includes cytokines IL-6, IL-8, and TGF-β, which create a pro-inflammatory and immunosuppressive tumor microenvironment. Targeting SASP components can mitigate the pro-tumorigenic effects of senescent cells [35]. Senomorphic compounds may play a role in suppressing the SASP, however, this class of compounds has not entered clinical trials. For example, sirtulin 1 (SIRT1), a nuclear NAD+ dependent histone deacetylase has been shown to suppress the SASP through epigenetic regulation [17, 36]. A notable disadvantage to senomorphs is they require chronic use to maintain their suppressive effect on the SASP.

In summary, senolytic agents can induce apoptosis in senescent cells, and senomorphic agents can modulate the SASP to reduce its pro-tumorigenic effects, demonstrating their potential therapeutic effects. Further investigation is needed to validate these approaches and establish their efficacy in patients with ULs.

## Discussion

Senescence plays a significant role in conditions featuring pathologic fibrosis. Senescent cells are found in keloid scars, pulmonary fibrosis, atherosclerosis, and leiomyomas. Leiomyomas feature a dysregulated state of fibrosis. Based on current data, the senescent cells found in leiomyomas exhibited similar characteristics to senescent cells in other fibrotic conditions such as elevated levels of senescent markers compared to matched normal tissue, increase extracellular matrix, and response to senolytic treatments.

A defining feature of uterine leiomyomas (ULs) is the presence of an excessive and disordered extracellular matrix (ECM). Studies have shown a connection between ECM and senescent cells. Increased ECM deposition can increase the number of cells undergoing senescence and senescent cells increase ECM deposition through SASPs [6]. Matrix metalloproteinases (MMPs) are important SASPs, and prior studies show that MMPs play an important role in the remodeling of extracellular matrix, promoting rapid growth in leiomyomas [33, 37]. Other SASPs of relevance to leiomyomas are interleukin-6 (IL-6) and transforming growth factor-β (TGF-β) which are known mediators of fibrosis. Decreased SIRT1 expression is associated with increased levels of the pro-inflammatory cytokines IL-6 and IL-8 in fibroblast cell lines obtained from human lung and foreskin, contributing to the senescent phenotype and the tumor microenvironment [38]. In pulmonary fibrosis, TGF-β expression was increased by 250-fold in senescent cell media compared to control [39]. The relationship between senescence and specific SASPs within leiomyomas needs to be further evaluated because SASP expression is dependent on the location of the senescent cell, cause of senescence, hormone exposure, and how long it has been present [40].

Within the studies reviewed, several mechanistic pathways remain poorly characterized. Oh et al. reported shorter telomeres in ULs compared to myometrium [25]. The alterations in telomere length might be due to a relative increase in the growth rate within ULs compared to myometrium and reflective of replicated senescence. Alternatively, the mutations within the ULs may be recognized by DNA repair mechanisms and undergo senescence to protect against proliferation of abnormal cells. The specific mechanisms by which telomere shortening is associated with the induction of cellular senescence in ULs remains underexplored. Markowski et al. [24] reported a 10-fold increase in expression of p14ARF in ULs compared to myometrium. The expression of p19ARF, the murine analog to human p14ARF, increased to 16.6-fold in *HMGA2* overexpressing 12q14 ~ 15 ULs compared to matched myometrium [31]. However, another study by Markowski et al. [28] noted that *HMGA2* overexpression was correlated with decreased p14ARF expression. Thus, the relationship between *HMGA2* expression and p14ARF in ULs is likely complex and requires further investigation.

The variability of senescence in ULs by race have not been directly evaluated. Other racial differences in ULs, however, have been well-studied: Black women are more likely to develop leiomyomas at an earlier age, with greater tumor burden, higher likelihood of anemia, and severe pelvic pain compared to White women [41]. Davis et al. [42] reported that leiomyomas in older Black women compared to older White women tend to have higher growth rates and were associated with differences in apoptosis-related genes like *BCL2* and *CASP3* and signaling pathways like NF-κB. These differences in apoptosis regulation pathways suggest possible differences in the regulation of senescence pathways between these two races, however, this was not explicitly reported. Additionally, Li et al. [41] found that leiomyomas from Black women have a higher rate of *MED12* mutations compared to White women. *MED12* mutations play a crucial role in the pathogenesis of ULs by disrupting cyclin C-CDK8/9 kinase activity, thus increasing levels of AKT signaling [43]. The AKT pathway subsequently promotes cellular proliferation and survival and inactivates cellular senescence pathways. Overall, this finding may suggest that Black women have lower degrees of cellular senescence due to higher burden of *MED12* mutations compared to White women. Li et al. [41] also investigated markers of oxidative stress which modulate several pathways including reactive oxygen species (ROS), hypoxia, and oxidative phosphorylation pathways. In their study, samples derived from Black patients demonstrated a higher oxidative stress burden across these pathways, including higher levels of oxidative stress markers, such as 8-hydroxyguanosine (8-OHdG) and heme oxygenase-1 (HO-1), compared to those from White patients. Higher levels of oxidative stress increase oxidative DNA damage, which in turn, may promote pro-senescent pathways [33]. Overall, the impact of *MED12* and oxidative stress on cellular senescence in ULs in Black women appear to contradict one another. Regardless, these findings suggest that ULs in Black women carry distinct genetic and molecular signatures that subsequently affect their senescence profiles.

Senolytics are emerging as treatment options for several fibrotic conditions besides ULs. Justice et al. [44]. conducted an open-label clinical trial evaluating intermittent use of senolytic drugs, dasatinib and quercetin orally for a total of nine doses to treat idiopathic pulmonary fibrosis. Patients who completed the trial had both clinical and significantly improved 6-min walk distance, 4-m gait speed, and chair-stands time (*p* <.05). However pulmonary function, clinical chemistries, frailty index (FI-LAB), and reported health were unchanged. SASP factors were evaluated by blood draw and found a decrease in IL-6 and MMP-7, though this was not statistically significant and perhaps related to the study being inadequately powered. The side effects reported were mild overall, however there was one incident of pneumonia [44]. Darmawan et al. [11] evaluated dasatinib injections within xenotransplanted keloid lesions and found a significant decrease in the weight of the keloid tissue, amount of procollagen and p16 expression compared to control. Senolytics reduced the number of senescent cells and increased the expression of pro-apoptotic markers in ULs as reported by Xie et al. [13], Xu et al. [33], and Markowski et al. [24, 28] and could be further evaluated as a new potential therapeutic drug class for management of ULs.

Due to limited data on the effects of senolytics on healthy cells, one potential therapeutic approach would be the direct administration into leiomyomas under transvaginal ultrasound guidance. Direct injection is possible, as reported in a phase 1 clinical trial conducted by Singh et al. [45] where collagenase was injected into leiomyomas under ultrasound guidance. In treated fibroids, collagen content decreased, but no increase in apoptotic cells was found. Therefore, a combination treatment of collagenase and senolytic might target both the excessive ECM and cells that augment ECM deposition. An alternative route of delivery of senolytics could be at the time of myomectomy to target senescent cells that remain at the surgical site, which are postulated to contribute to the 25% recurrence rate of leiomyomas [46].

Overall, there is limited research on the influence of senescent cells on leiomyomas. Due to limited research, all studies were included that related to the topic regardless of the risk of bias. Many studies did not adjust for confounding variables when assessing for senescence, such as the patient’s age or genetic phenotype of the fibroid. Leiomyomas are heterogenous in nature, and while some studies included tumor size and karyotype, most studies did not include patient demographics or FIGO classification. Studies in the future would benefit from standardization of results. The current lack of standardization between studies contributed to the limited sub-analysis.

## Conclusion

Leiomyomas are a fibrotic condition characterized by the presence of senescent cells, which are also observed in other fibrotic conditions such as keloids and pulmonary fibrosis. Leiomyomas exhibit increased expression of senescent markers compared to matched normal tissues, increased extracellular matrix, and have variable responses to senolytic treatments. However, the current evidence is limited, and further research is needed to elucidate how senescent cells in leiomyomas contribute to their growth, recurrence and impact therapeutic options.

## Supplementary Information

Below is the link to the electronic supplementary material.

Supplementary Material 1 (DOCX 13.4 KB)

Supplementary Material 2 (DOCX 11.1 KB)

## Data Availability

The data that support the findings of this study were derived from the following resources available in the public domain: PubMed, Embase, Scopus, and Web of Science.

## Code Availability

N/A.

## References

[1] Owen C, Armstrong AY. Clinical management of leiomyoma. Obstet Gynecol Clin North Am. 2015;42(1):67–85. 10.1016/j.ogc.2014.09.009. 25681841 10.1016/j.ogc.2014.09.009

[2] Holdsworth-Carson SJ, Zhao D, Cann L, Bittinger S, Nowell CJ, Rogers PAW. Differences in the cellular composition of small versus large uterine fibroids. Reproduction. 2016;152(5):467–80. 10.1530/REP-16-0216. 27528771 10.1530/REP-16-0216

[3] Stewart EA. Uterine fibroids. Lancet. 2001;357(9252):293–8. 10.1016/S0140-6736(00)03622-9. 11214143 10.1016/S0140-6736(00)03622-9

[4] Neumann B, Singh B, Brennan J, Blanck J, Segars JH. The impact of fibroid treatments on quality of life and mental health: a systematic review. Fertil Steril. 2024;121(3):400-425. 10.1016/j.fertnstert.2024.01.021. 10.1016/j.fertnstert.2024.01.021 PMC11140829 38246400

[5] Hazimeh D, Coco A, Casubhoy I, Segars J, Singh B. The annual economic burden of uterine fibroids in the United States (2010 versus 2022): a comparative cost-analysis. Reprod Sci. 2024. 10.1007/s43032-024-01727-0. 10.1007/s43032-024-01727-0 PMC12720250 39455488

[6] Levi N, Papismadov N, Solomonov I, Sagi I, Krizhanovsky V. The ECM path of senescence in aging: components and modifiers. FEBS J. 2020;287(13):2636–46. 10.1111/febs.15282. 32145148 10.1111/febs.15282

[7] Jun J-I, Lau LF. The matricellular protein CCN1 induces fibroblast senescence and restricts fibrosis in cutaneous wound healing. Nat Cell Biol. 2010;12(7):676–85. 10.1038/ncb2070. 20526329 10.1038/ncb2070 PMC2919364

[8] Schmitt CA, Wang B, Demaria M. Senescence and cancer — role and therapeutic opportunities. Nat Rev Clin Oncol. 2022. 10.1038/s41571-022-00668-4. 36045302 10.1038/s41571-022-00668-4 PMC9428886

[9] Reynolds LE, Maallin S, Haston S, Martinez-Barbera JP, Hodivala-Dilke KM, Pedrosa AR. Effects of senescence on the tumour microenvironment and response to therapy. FEBS J. 2024;291:2306-2319. 10.1111/febs.16984. 10.1111/febs.16984 37873605

[10] Saab R. Senescence and pre-malignancy: how do tumors progress? Semin Cancer Biol. 2011;21(6):385-391. 10.1016/j.semcancer.2011.09.013. 10.1016/j.semcancer.2011.09.013 21982725

[11] Darmawan CC, Hur K, Kusumaningrum N, Chung JH, Lee S-H, Mun J-H. Dasatinib attenuates fibrosis in keloids by decreasing senescent cell burden. Acta Derm Venereol. 2023;103:adv4475. 10.2340/actadv.v103.4475. 37021598 10.2340/actadv.v103.4475 PMC10108619

[12] Xu X, et al. Inactivation of AKT induces cellular senescence in uterine leiomyoma. Endocrinology. 2014;155(4):1510–9. 10.1210/en.2013-1929. 24476133 10.1210/en.2013-1929 PMC3959594

[13] Xie J, et al. Application of ex-vivo spheroid model system for the analysis of senescence and senolytic phenotypes in uterine leiomyoma. Lab Invest. 2018;98(12):1575–87. 10.1038/s41374-018-0117-5. 30206313 10.1038/s41374-018-0117-5 PMC6265265

[14] Wagner K-D, Wagner N. The senescence markers p16INK4A, p14ARF/p19ARF, and p21 in organ development and homeostasis. Cells. 2022;11(12):1966. 10.3390/cells11121966. 35741095 10.3390/cells11121966 PMC9221567

[15] Prasanna PG, et al. Therapy-induced senescence: opportunities to improve anticancer therapy. JNCI. 2021;113(10):1285–1298. 10.1093/jnci/djab064. 10.1093/jnci/djab064 PMC8486333 33792717

[16] Fakhri S, Zachariah Moradi S, DeLiberto LK, Bishayee A. Cellular senescence signaling in cancer: a novel therapeutic target to combat human malignancies. Biochem Pharamcol. 2022;199:114989. 10.1016/j.bcp.2022.114989. 10.1016/j.bcp.2022.114989 35288153

[17] Lagoumtzi SM, Chondrogianni N. Senolytics and senomorphics: natural and synthetic therapeutics in the treatment of aging and chronic diseases. Free Radic Biol Med. 2021;171:169–90. 10.1016/j.freeradbiomed.2021.05.003. 33989756 10.1016/j.freeradbiomed.2021.05.003

[18] Billimoria R, Bhatt P. Senescence in cancer: advances in detection and treatment modalities. Biochem Pharmacol. Biochem Pharmacol. 2023;215:115739. 10.1016/j.bcp.2023.115739. 10.1016/j.bcp.2023.115739 37562510

[19] Lucas V, Cavadas C, Aveleira CA. Cellular senescence: from mechanisms to current biomarkers and senotherapies. Pharmacol Rev. 2023;75(4):675–713. 10.1124/pharmrev.122.000622. 36732079 10.1124/pharmrev.122.000622

[20] Page MJ et al. Mar., The PRISMA 2020 statement: an updated guideline for reporting systematic reviews. BMJ. 2021;372:n71. 10.1136/bmj.n71. 10.1136/bmj.n71 PMC8005924 33782057

[21] Stang A. Critical evaluation of the Newcastle-Ottawa scale for the assessment of the quality of nonrandomized studies in meta-analyses. Eur J Epidemiol. 2010;25(9):603–5. 10.1007/s10654-010-9491-z. 20652370 10.1007/s10654-010-9491-z

[22] Laser J, Lee P, Wei J-J. Cellular senescence in usual type uterine leiomyoma. Fertil Steril. 2010;93(6):2020–6. 10.1016/j.fertnstert.2008.12.116. 19217096 10.1016/j.fertnstert.2008.12.116

[23] Yang NC, Hu ML. The limitations and validities of senescence associated-β- galactosidase activity as an aging marker for human foreskin fibroblast Hs68 cells. Exp Gerontol. 2005;40(10):813–9. 10.1016/j.exger.2005.07.011. 16154306 10.1016/j.exger.2005.07.011

[24] Markowski DN, et al. Fibroid explants reveal a higher sensitivity against MDM2-inhibitor nutlin-3 than matching myometrium. BMC Womens Health. 2012;12(1):2. 10.1186/1472-6874-12-2. 22233735 10.1186/1472-6874-12-2 PMC3276409

[25] Oh B-K, Choi Y, Choi JS. Telomere shortening and expression of TRF1 and TRF2 in uterine leiomyoma. Mol Med Rep. 2021;24(2):606. 10.3892/mmr.2021.12243. 34184077 10.3892/mmr.2021.12243

[26] Xie J, Ubango J, Ban Y, Chakravarti D, Kim JJ, Wei J. Comparative analysis of AKT and the related biomarkers in uterine leiomyomas with MED12, HMGA2, and FH mutations. Genes Chromosomes Cancer. 2018;57(10):485–94. 10.1002/gcc.22643. 29790226 10.1002/gcc.22643 PMC6128746

[27] Ferrero H. HMGA2 involvement in uterine leiomyomas development through angiogenesis activation. Fertil Steril. 2020;114(5):974–5. 10.1016/j.fertnstert.2020.07.044. 32943224 10.1016/j.fertnstert.2020.07.044

[28] Markowski DN, Bartnitzke S, Belge G, Drieschner N, Helmke BM, Bullerdiek J. Cell culture and senescence in uterine fibroids. Cancer Genet Cytogenet. 2010;202(1):53–7. 10.1016/j.cancergencyto.2010.06.010. 20804922 10.1016/j.cancergencyto.2010.06.010

[29] Park BH, Vogelstein B, Tumor suppressor genes. In: Kufe DW, Pollock RE, Weichselbaum RR, Bast Jr RC, Gansler TS, Holland JF, Frei III E, editors. Holland-Frei cancer medicine. 6th edition. Hamilton, ON: BB Decker; 2003.

[30] Markowski DN, Von Ahsen I, Nezhad MH, Wosniok W, Helmke BM, Bullerdiek J. HMGA2 and the p19Arf-TP53-CDKN1A axis: a delicate balance in the growth of uterine leiomyomas. Genes Chromosomes Cancer. 2010;49(8):661–8. 10.1002/gcc.20777. 20544840 10.1002/gcc.20777

[31] Markowski DN, von Ahsen I, Nezhad MH, Wosniok W, Helmke BM, Bullerdiek J. HMGA2 and the p19 Arf -TP53‐CDKN1A axis: a delicate balance in the growth of uterine leiomyomas. Genes Chromosomes Cancer. 2010;49(8):661–8. 10.1002/gcc.20777. 20544840 10.1002/gcc.20777

[32] Galindo LJ, et al. HMGA2 and MED12 alterations frequently co-occur in uterine leiomyomas. Gynecol Oncol. 2018;150(3):562–8. 10.1016/j.ygyno.2018.07.007. 30017537 10.1016/j.ygyno.2018.07.007

[33] Xu X, Kim JJ, Li Y, Xie J, Shao C, Wei J-J. Oxidative stress-induced miRNAs modulate AKT signaling and promote cellular senescence in uterine leiomyoma. J Mol Med. 2018;96(10):1095–106. 10.1007/s00109-018-1682-1. 30097674 10.1007/s00109-018-1682-1 PMC6135677

[34] Rudin CM, et al. Phase II study of single-agent navitoclax (ABT-263) and biomarker correlates in patients with relapsed small cell lung cancer. Clin Cancer Res. 2012;18(11):3163–9. 10.1158/1078-0432.CCR-11-3090. 22496272 10.1158/1078-0432.CCR-11-3090 PMC3715059

[35] Saad EE, Michel R, Borahay MA. Senescence-associated secretory phenotype (SASP) and uterine fibroids: association with PD-L1 activation and collagen deposition. Ageing Res Rev. 2024;97:102314. 10.1016/j.arr.2024.102314. 10.1016/j.arr.2024.102314 PMC11181954 38670462

[36] Dickson BC, Riddle ND, Brooks JS, Pasha TL, Zhang PJ. Sirtuin 1 (SIRT1): a potential immunohistochemical marker and therapeutic target in soft tissue neoplasms with myoid differentiation. Hum Pathol. 2013;44(6):1125–30. 10.1016/j.humpath.2012.10.001. 23332867 10.1016/j.humpath.2012.10.001

[37] Onishi K, Zhang J, Blanck JF, Singh B. A systematic review of matrix metalloproteinases as potential biomarkers for uterine fibroids. F&S Reviews. 2022;3(4):227–41. 10.1016/j.xfnr.2022.07.003.

[38] Hayakawa T, et al. SIRT1 suppresses the senescence-associated secretory phenotype through epigenetic gene regulation. PLoS One. 2015. 10.1371/journal.pone.0116480. 25635860 10.1371/journal.pone.0116480 PMC4312089

[39] Schafer MJ, et al. Cellular senescence mediates fibrotic pulmonary disease. Nat Commun. 2017;8(1):14532. 10.1038/ncomms14532. 28230051 10.1038/ncomms14532 PMC5331226

[40] Tripathi U, Misra A, Tchkonia T, Kirkland JL. Impact of senescent cell subtypes on tissue dysfunction and repair: importance and research questions. Mech Ageing Dev. 2021;198:111548. 10.1016/j.mad.2021.111548. 34352325 10.1016/j.mad.2021.111548 PMC8373827

[41] Li Y, McNally RP, Feng Y, Kim JJ, Wei JJ. Racial differences in transcriptomics and reactive oxygen species burden in myometrium and leiomyoma. Hum Reprod. 2023;38(4):609–20. 10.1093/humrep/dead020. 10.1093/humrep/dead020 PMC10068273 36749068

[42] Davis BJ, Risinger JI, Chandramouli GVR, Bushel PR, Baird DD, Peddada SD. Gene expression in uterine leiomyoma from tumors likely to be growing (from Black women over 35) and tumors likely to be non-growing (from White women over 35). PLoS One. 2013;8(6). 10.1371/journal.pone.0063909. 10.1371/journal.pone.0063909 PMC3681799 23785396

[43] Amendola ILS, Spann M, Segars J, Singh B. The mediator complex subunit 12 (MED-12) gene and uterine fibroids: a systematic review. Reprod Sci. 2024;31(2):291–308. 10.1007/s43032-023-01297-7. 37516697 10.1007/s43032-023-01297-7

[44] Justice JN, et al. Senolytics in idiopathic pulmonary fibrosis: results from a first-in-human, open-label, pilot study. EBioMedicine. 2019;40:554–63. 10.1016/j.ebiom.2018.12.052. 30616998 10.1016/j.ebiom.2018.12.052 PMC6412088

[45] Singh B, et al. A phase I clinical trial to assess safety and tolerability of injectable collagenase in women with symptomatic uterine fibroids. Reprod Sci. 2021;28(9):2699–709. 10.1007/s43032-021-00573-8. 33914296 10.1007/s43032-021-00573-8 PMC8346429

[46] Hartmann KE, et al. Management of uterine fibroids. Comparative effectiveness review volume 195. Rockville, MD: Agency for Healthcare Research and Quality; 2017. 30789683

