# Supplementary material for: A Systematic Review of the Role of Senescent Cells in Uterine Leiomyomas: Deciphering Molecular Pathways and Exploring Therapeutic Prospects
Source: Reprod Sci. 2026 May 5;33(5):853–63. doi: 10.1007/s43032-026-02075-x (PMC13230283; doi:10.1007/s43032-026-02075-x)
Supplement: Supplementary file 1 — Supplementary Material 1 (DOCX 13.4 KB) [file 43032_2026_2075_MOESM1_ESM.docx]

Appendix 1 Search Terms by Set of Concepts

| Database | Concept | Search Statement |
| --- | --- | --- |
| PubMed Search | Senescence and Leiomyomas | (("Cellular Senescence"[Mesh] OR "senescence"[tiab] OR "senescent"[tiab] OR "senescent cell*"[tiab] OR "cell aging"[tiab] OR "cellular aging"[tiab] OR "cell ageing"[tiab] OR "cellular ageing"[tiab] OR "immunosenescence"[tiab] OR "senesce"[tiab])) AND (("Leiomyoma"[Mesh] OR "leiomyoma"[tiab] OR "leiomyomas"[tiab] OR "leiomyomata"[tiab] OR "leiomyomatas"[tiab] OR "fibromyoma"[tiab] OR "fibromyomas"[tiab] OR "myoma"[tiab] OR "myomas"[tiab] OR "myomatosis"[tiab] OR (("fibroid"[tiab] OR "fibroids"[tiab]) AND ("uterus"[tiab] OR "uterine"[tiab] OR "uteri"[tiab] OR "myometrium"[tiab] OR "Myometrium"[Mesh])) OR "ULM"[tiab])) |
| Embase Search | Senescence and Leiomyomas | ('cell aging'/exp OR 'senescence':ti,ab,kw OR 'senescent':ti,ab,kw OR 'senescent cell*':ti,ab,kw OR 'cell aging':ti,ab,kw OR 'cellular aging':ti,ab,kw OR 'cell ageing':ti,ab,kw OR 'cellular ageing':ti,ab,kw OR 'immunosenescence':ti,ab,kw OR 'senesce':ti,ab,kw) AND ('leiomyoma'/exp OR 'leiomyoma':ti,ab,kw OR 'leiomyomas':ti,ab,kw OR 'leiomyomata':ti,ab,kw OR 'leiomyomatas':ti,ab,kw OR 'fibromyoma':ti,ab,kw OR 'fibromyomas':ti,ab,kw OR 'myoma':ti,ab,kw OR 'myomas':ti,ab,kw OR 'myomatosis':ti,ab,kw OR 'ulm':ti,ab,kw OR (('fibroid':ti,ab,kw OR 'fibroids':ti,ab,kw) AND ('uterus':ti,ab,kw OR 'uterine':ti,ab,kw OR 'uteri':ti,ab,kw OR 'myometrium':ti,ab,kw OR 'myometrium'/exp))) |
| Web of Science | Senescence and Leiomyomas | TS=("leiomyoma" OR "leiomyomas" OR "leiomyomata" OR "leiomyomatas" OR "fibromyoma" OR "fibromyomas" OR "myoma" OR "myomas" OR "myomatosis" OR "ulm" OR (( "fibroid" OR "fibroids") AND ("uterus" OR "uterine" OR "uteri" OR "myometrium")) ) AND TS=("cellular senescence" OR "senescence" OR "senescent" OR "senescent cell*" OR "cell aging" OR "cellular aging" OR "cell ageing" OR "cellular ageing" OR "immunosenescence" OR "senesce") |
| Scopus | Senescence and Leiomyomas | TITLE-ABS-KEY ( "cellular senescence" OR "senescence" OR "senescent" OR "senescent cell*" OR "cell aging" OR "cellular aging" OR "cell ageing" OR "cellular ageing" OR "immunosenescence" OR "senesce" ) AND TITLE-ABS-KEY ( "leiomyoma" OR "leiomyomas" OR "leiomyomata" OR "leiomyomatas" OR "fibromyoma" OR "fibromyomas" OR "myoma" OR "myomas" OR "myomatosis" OR "ulm" OR ( ( "fibroid" OR "fibroids" ) AND ( "uterus" OR "uterine" OR "uteri" OR "myometrium" ) ) ) |
