# Supplementary material for: A Systematic Review of the Role of Senescent Cells in Uterine Leiomyomas: Deciphering Molecular Pathways and Exploring Therapeutic Prospects
Source: Reprod Sci. 2026 May 5;33(5):853–63. doi: 10.1007/s43032-026-02075-x (PMC13230283; doi:10.1007/s43032-026-02075-x)
Supplement: Supplementary file 2 — Supplementary Material 2 (DOCX 11.1 KB) [file 43032_2026_2075_MOESM2_ESM.docx]

Appendix 2 Criteria for Inclusion and Exclusion of Studies

|  | Inclusion Criteria | Exclusion Criteria |
| --- | --- | --- |
| Population | Uterine fibroids from human subjects | Animal studies |
| Results | Evaluate senescence pathways or use senolytics or senomorphics | Does not evaluate senescence |
| Study Design | Randomized control trials, observational studies | Case study, case report, case control, literature reviews |
